# Women receive more trials of noninvasive ventilation for acute respiratory failure than men: a nationwide population-based study

**DOI:** 10.1186/cc10323

**Published:** 2011-07-25

**Authors:** Hsiu-Nien Shen, Chin-Li Lu, Hsi-Hsing Yang

**Affiliations:** 1 Department of Intensive Care Medicine, Chi Mei Medical Center, No. 901 Zhonghua Road, Yongkang Dist., Tainan City 710, Taiwan; 2 Department of Medical Research, Chi Mei Medical Center, No. 901 Zhonghua Road, Yongkang Dist., Tainan City 710, Taiwan

## Abstract

**Introduction:**

Previous studies in western countries have observed that women are less likely than men to receive intensive care and mechanical ventilation (MV). We aimed to investigate whether the gender difference also exists in Asian populations and in the provision of different types of MV including invasive (INV) and noninvasive ventilation (NIV).

**Methods:**

We analyzed all adult hospital patients between 2005 and 2007 in the claims data from 1,000,000 randomly selected people in the Taiwan National Health Insurance Research Database. NIV-only was defined as patients receiving NIV as the only ventilator treatment during hospitalization. Gender difference was assessed using multivariable analyses with/without considering a hospital cluster effect by generalized estimating equations models. Subgroup analyses for gender difference in NIV use were performed using propensity score matching method.

**Results:**

Of the 128,327 patients enrolled, 53.8% were men, 9.2% received intensive care and 5.2% used MV. After adjusting for potential confounders, women were less likely than men to receive intensive care (adjusted odds ratio [aOR] 0.77, 95% confidence interval [CI] 0.73-0.82) and MV (aOR 0.84, 95% CI 0.78-0.91). Among MV patients, 6.8% received NIV-only; the proportion of which was higher in women than in men (8.6% vs. 5.7%, *P *< 0.001). After controlling for confounders and a cluster effect, women remained more likely to receive NIV-only (aOR 1.61, 95% CI 1.32-1.96). Subgroup analyses showed that patients with underlying congestive heart failure (CHF) had the highest difference in the provision of NIV-only (female-to-male aOR 2.76, 95% CI 1.38-5.53). A hospital cluster effect on the gender difference in NIV use was found in patients with diseases other than chronic obstructive pulmonary disease and CHF.

**Conclusions:**

Gender differences existed not only in the provision but also in the types of MV. Further studies are needed to understand why gender differences occur.

## Introduction

Studies in western countries have shown that gender differences exist in the process of care for critically ill patients [1-3]. For example, women are found to be less likely than men to receive intensive care [1,2] and invasive treatments including mechanical ventilation (MV) [1-3]. The suboptimal care might contribute to an observed excess mortality in women [2]. Whether these differences exist in Asian populations remains unknown. Besides, the influence of gender on the provision of different types of MV, that is, invasive ventilation (INV) and noninvasive ventilation (NIV), has not been investigated.

NIV provides ventilator support via a nose/face mask without using an invasive artificial airway for patients with acute respiratory failure (ARF). The use of NIV has been shown to reduce intubation and mortality in ARF patients with acute pulmonary edema and underlying chronic obstructive pulmonary disease (COPD) [4-7]. In diseases other than COPD and acute pulmonary edema, NIV is also increasingly used with variable success rates [8-12]. The utilization rates of NIV in patients with ARF range from 16% to 24% in developed countries [12-14]. But these data are underestimated because NIV is increasingly initiated outside the ICU setting [14,15].

Women may be more likely to receive NIV if they are less likely than men to receive invasive treatments such as INV [1-3]. In addition, a higher perception of breathlessness in women [16,17] may increase the chance of receiving an NIV trial for "shortness of breath" because strict criteria are usually lacking in clinical practices [9]. In Taiwan, the National Health Insurance Research Database (NHIRD) covers nearly all (99%) claims for its population of more than 22 million. It has been used extensively in various studies [18-20] and can provide real world experiences of NIV use both outside and inside the ICU. Therefore, we conducted this study, based on the NHIRD, to analyze the nationwide utilization of NIV in Taiwan and to assess whether gender differences exist not only in the provision of intensive care and MV but also in the provision of different types of MV for patients with ARF. We hypothesized that women are less likely than men to receive intensive care and MV during hospitalization but receive more trials of NIV for ARF and that the gender difference in NIV use varies among subgroups of patients with underlying COPD, congestive heart failure (CHF) and other diseases.

## Materials and methods

### Database

In Taiwan, a compulsory and universal National Health Insurance (NHI) program was initiated by the government in 1995 [18]. With the exception of prison inmates, all citizens are enrolled in the program. Patients were drawn from the NHIRD, released for research purposes by the National Health Research Institute, Taipei, Taiwan. The NHIRD provided encrypted patient identification numbers, sex, birthday, dates of admission and discharge, medical institutions providing the services, the *ICD*-9-CM (*International Classification of Diseases, Ninth Revision, Clinical Modification*) codes of diagnoses (up to five) and procedures (up to five), outcome at hospital discharge (recovered, died or transferred out), order codes, and the fees charged to patients.

### Study sample

The study cohort was drawn from a subset of the NHIRD, the Longitudinal Health Insurance Database of 2005 (LHID-2005) [18]. One million beneficiaries, representing about 5% of the Taiwanese population enrolled in 2005, were selected using a simple random sampling method from the NHIRD and included in the LHID-2005 that contained all linked claims data of the cohort from 2005 to 2007. There were no significant differences in age and sex between the study cohort and the general population [18]. The study period spanned from 1 January, 2005 to 31 December, 2007. All adult (≥ 18 years old) patients were identified by linking through the hospitalization claims data. Patients were excluded if they were hospitalized for mental disorders (*ICD*-9-CM codes 290-319) or had diagnoses related to complications of pregnancy, childbirth or the puerperium (*ICD*-9-CM codes 630-679); the reasons for exclusion were reduced likeliness of receiving non-psychiatric acute medical treatment for the former and a lack of relevant male-related codes for the latter. Patients were also excluded if they were ventilator dependent in the respiratory wards or received negative pressure or high frequency oscillatory ventilation due to their chronic respiratory failure and/or receipt of uncommon mode of ventilation. To ensure the independence of observations, only the first-episode admissions were included in the analyses. Human Subjects Institutional Review Board Approval and informed consent were not needed because the study used an encrypted administrative database.

### Definition

Patients who received MV during acute care hospitalizations were designated as ARF [21]. INV-only was used to define those receiving INV as the only ventilator treatment and NIV-only was for those receiving only NIV during the same hospitalization. Because we could not verify the sequence, NIV/INV described those who received both NIV and INV during the same hospitalization. NIV was delivered using continuous positive airway pressure and/or bilevel positive airway pressure ventilation. The specific indications for MV or use of NIV could not be determined because the database did not provide such information. As claims reimbursement for use of MV was based on a per day basis, not per hour use, and only one claim can be filed for those receiving both INV and NIV on the same day, only the claim for the higher-cost INV was made. For example, if one ARF patient received NIV on day one but, due to NIV failure, required INV from day two to five, and then when the patient was weaned from INV, received NIV again on day six for post-extubation respiratory distress, it would show "NIV for two days" (i.e., day one and six) and "INV for four days" (i.e., day two to five) on claims data. The use of NIV on day two would not be eligible for reimbursement.

Definitions of readmission, surgical conditions, and hospital mortality have been described previously [19].

### Measurements

Baseline characteristics of study subjects were examined, including age, gender, medical/surgical conditions, hospital levels (medical centers with > 500 beds, regional hospitals with 250 to 500 beds, and district hospitals with 20 to 249 beds), prevalence of selected comorbid conditions (including COPD, CHF, cerebrovascular disease, and cancer), Charlson Comorbidity Index [22,23], principal diagnoses, and occurrence of acute organ dysfunction (as a measure of disease severity) [19]. Outcome measures included use and types of MV (including INV-only, NIV-only, and NIV/INV), ICU admission, duration of MV, length of stays, and hospital mortality [19]. COPD was defined as *ICD*-9-CM codes 490 to 496 (excluding 493 for asthma). Definitions of CHF, cerebrovascular disease, and cancer were based on the Charlson Comorbidity Index [22,23], which is a weighted summary measure of clinically important concomitant diseases that has been adapted for use with *ICD*-9-CM coded administrative databases.

### Statistics

Continuous variables were described as median (interquartile range (IQR)) and compared by the Mann-Whitney U test; discrete ones were expressed as counts or percentages and analyzed by the chi-square test. To examine the effects of gender on various outcomes, we conducted multivariable logistic regression analyses adjusting for the baseline covariates, including age, surgical and selected comorbid conditions, Charlson Comorbidity Index, hospital levels, principal diagnoses and number of acute organ dysfunction. Additional covariates such as status of MV or intensive care were included as appropriate. Specifically, MV was included as an additional covariate when modeling the gender effect on the provision of intensive care and on hospital mortality of patients requiring intensive care. Conversely, intensive care status was included as an additional covariate when modeling the gender effect on the provision of MV for all patients and that of different types of MV for ARF patients as well as on hospital mortality of ARF patients. Resource uses (including duration of MV and length of stays) were compared using multivariable linear regression models adjusting for the baseline covariates, with the inclusion of MV and intensive care status as additional covariates when appropriate. Tolerance level was calculated to assess multicollinearity, which was defined as existing when the level was less than 0.1.

To further explore the effect of gender on the provision of different types of MV, we also applied the propensity score method [24-26] for ARF patients and for subgroups with underlying COPD, CHF, and others. Patients with COPD or CHF were selected because they were associated with increased use of NIV [4,5]. The propensity score method has been used to reduce bias and increase precision of estimates in observational research [24-26]. The propensity score, that is the probability of being a female patient, was estimated by a logistic regression model conditional on the baseline covariates and intensive care status. For all ARF patients and subgroups, men and women were matched one-to-one by the propensity score using the greedy-matching algorithm [26] and then analyzed in the logistic regression models to estimate the gender effect on the provision of NIV. Because hospitals tended to behave differently and outcomes of patients within the same hospitals were correlated, a hospital cluster effect might be present [27]. Therefore, the gender effect on the provision of NIV was reanalyzed using logistic Generalized Estimating Equations (GEE) models [27], specifying an exchangeable structure of a working correlation matrix to regress the correlated binary outcomes. The performance of the propensity score model was assessed by examining whether the baseline covariates of men and women were balanced after matching. Data analysis was performed using SPSS for Windows, version 17.0. (SPSS Inc., Chicago, IL, USA) and SAS software, version 9.1 (SAS Institute, Inc., Cary, NC, USA). Statistical significance was set at *P *< 0.05 (two-tailed).

## Results

**Figure 1:**
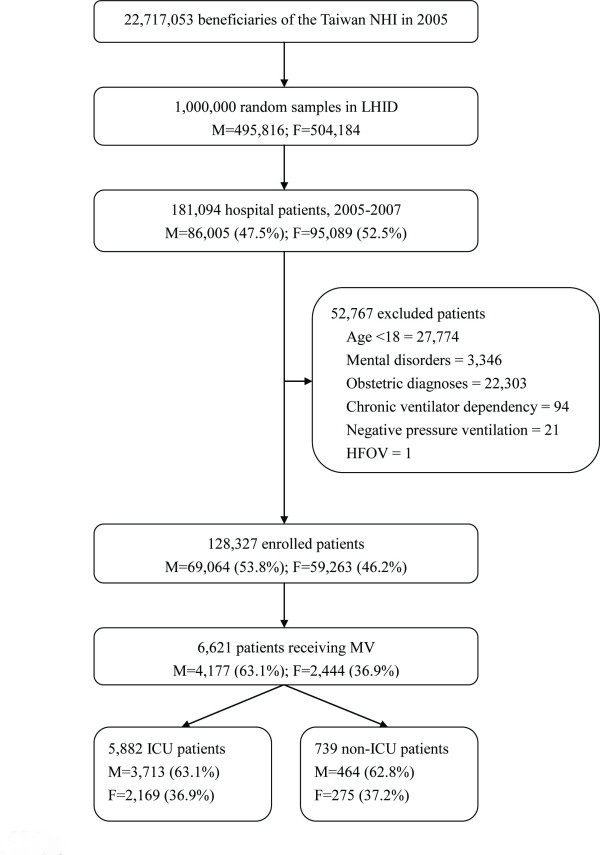
**Flow diagram of the study patients**. The same excluded patient may appear in different categories. F, female; HFOV, high frequency oscillatory ventilation; LHID, the Longitudinal Health Insurance Database of 2005; M, male; MV, mechanical ventilation; NHI, National Health Insurance.

**Figure 2:**
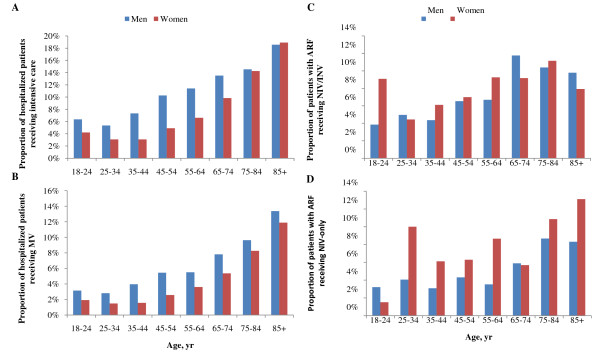
**Age-and-gender-specific proportional distributions of patients receiving (a) intensive care, (b) mechanical ventilation (MV), (c) noninvasive and invasive ventilation (NIV/INV) and (d) NIV-only**. Note: NIV-only was used to define those receiving NIV as the only ventilator treatment and NIV/INV was for those receiving both NIV and INV during the same hospitalization. ARF, acute respiratory failure.

During the three-year study period, we identified 128,327 patients. Of them, 53.8% were men, 9.2% had an ICU stay and 5.2% received MV (Figure 1). Characteristics of the study population are shown in Table 1. Women were less likely than men to receive intensive care (*P *< 0.001) and MV (*P *< 0.001). The differences were consistent across all age groups except those aged 85 years or older (Figures 2a and 2b). After adjusting for potential confounders (i.e., baseline and additional covariates as described in the Methods section), women remained less likely than men to receive intensive care (adjusted odds ratio (aOR) 0.77, 95% confidence interval (CI) 0.73-0.82) and MV (aOR 0.84, 95% CI 0.78-0.91).

**Table 1:** Characteristics of the study population (*n *= 128,327)

Variables	Men (*n *= 69,064)	Women (*n *= 59,263)
**Age, years**	54 (39-70)	55 (41-71)
**≥ 65**	34.1	35.3
**Surgical condition**	43.8	47.3
**Comorbidity**		
**Charlson Comorbidity Index**	0 (0-1)	0 (0-1)
**COPD**	4.7	2.1
**Congestive heart failure**	2.8	3.1
**Cerebrovascular disease**	7.3	6.2
**Cancer**	8.2	7.7
**Principal diagnoses**		
**Respiratory**	10.8	8.5
**Neurological**	3.3	4.1
**Cardiovascular**	14.2	11.7
**Digestive**	16.4	10.8
**Genitourinary**	8.8	13.6
**Metabolic/endocrine**	2.6	3.8
**Injury/poisoning**	19.5	15.9
**Others**	24.5	31.6
**No. of organ dysfunction**		
**0**	90.4	92.6
**1**	8.1	6.3
**2+**	1.5	1.1
**Mechanical ventilation**	6.0	4.1
**ICU admission**	10.6	7.5
**Hospital level**		
**Medical center**	34.0	35.4
**Regional hospital**	41.8	40.3
**District hospital**	24.2	24.3

Values are expressed as median (interquartile range) or percentages.

COPD, chronic obstructive pulmonary disease.

Distributions of different types of MV in ARF patients are shown in Table 2. The age-and-gender-specific distribution of ARF patients receiving NIV is shown in Figures 2c and 2d. Among ARF patients, 6.8% received NIV-only, of which a higher proportion was in women than in men (Table 2).

**Table 2:** Distributions of different types of mechanical ventilation in patients with acute respiratory failure

Variables	No	**INV-only**, %	**NIV-only**, %	**NIV/INV**, %	*P *Value
**Total**	6,621	84.6	6.8	8.6	
**Sex**					< 0.001
**Men**	4,177	85.9	5.7	8.4	
**Women**	2,444	82.4	8.6	9.0	
**Age**					< 0.001
**< 65 years**	2,917	88.9	4.8	6.3	
**≥ 65 years**	3,704	81.3	8.3	10.4	
**Surgical condition**					< 0.001
**Yes**	3,594	87.5	3.5	9.0	
**No**	3,027	81.2	10.6	8.1	
**Charlson Comorbidity index**					< 0.001
**0**	2,158	88.3	4.4	7.3	
**1**	2,076	83.9	7.0	9.1	
**2**	1,258	81.4	9.6	9.0	
**3+**	1,129	82.4	7.7	9.9	
**Comorbid conditions**					
**COPD**					< 0.001
**Yes**	533	66.2	16.7	17.1	
**No**	6088	86.2	5.9	7.9	
**Congestive heart failure**					< 0.001
**Yes**	573	74.9	12.2	12.9	
**No**	6,048	85.5	6.3	8.2	
**Cerebrovascular disease**					< 0.001
**Yes**	1,118	89.6	3.2	7.2	
**No**	5,503	83.6	7.5	8.9	
**Cancer**					0.001
**Yes**	946	82.2	9.5	8.2	
**No**	5,675	85.0	6.3	8.7	
**Principal diagnoses**					< 0.001
**Respiratory**	1,395	76.3	12.4	11.3	
**Non-respiratory**	5,220	86.9	5.2	7.9	
**No. of organ dysfunction**					0.011
**1**	5,173	83.8	7.5	8.7	
**2**	1,198	86.9	4.5	8.6	
**3+**	250	90.4	2.4	7.2	
**ICU admission**					< 0.001
**Yes**	5,882	87.5	3.1	9.5	
**No**	739	61.8	36.3	1.9	
**Hospital level**					< 0.001
**Medical center**	2,861	81.0	7.7	11.4	
**Regional hospital**	2,802	86.3	6.2	7.5	
**District hospital**	958	90.6	5.6	3.8	

COPD, chronic obstructive pulmonary disease; INV-only, use of invasive ventilation (INV) as the only ventilator treatment; NIV-only, use of noninvasive ventilation (NIV) as the only ventilator treatment; NIV/INV, use of both NIV and INV before or after each other.

Characteristics, resource use, and outcomes of ARF patients stratified by gender and intensive care status are shown in Table 3. Among ARF patients, women were older than men inside or outside the ICU, and women being cared for inside the ICU had fewer surgical conditions. The distribution patterns of selected comorbid conditions were different in ICU patients of both genders but similar in those being cared for outside the ICU. But the distribution of principal diagnoses, number of organ dysfunction, and hospital-level patterns were comparable in both genders. Outcomes including the proportion of ICU admission (88.9% vs. 88.7%, *P *= 0.858), duration of MV, length of stays, and hospital mortality were similar between men and women. Among ARF patients receiving NIV (including NIV-only and NIV/INV), duration of NIV was significantly longer in women than in men (median three days, IQR one to five days vs. two days, IQR one to four days, respectively: crude and adjusted *P *= 0.025 and 0.043, respectively). The differences became insignificant when durations of NIV in patients receiving NIV-only and NIV/INV were calculated separately.

**Table 3:** Characteristics, resource use and outcomes of patients with acute respiratory failure by gender and intensive care status (*n *= 6,621)

Variables	With intensive care			Without intensive care		
	**Men (*n *= 3,713)**	**Women (*n *= 2,169)**	***P *Value**	**Men (*n *= 464)**	**Women (*n *= 275)**	***P *Value**

**Age, years**	65 (49-77)	71 (56-80)	< 0.001	70 (52-79)	72 (57-82)	0.032
**≥ 65**	50.9	62.9	< 0.001	59.9	62.2	0.542
**Surgical condition**	57.7	54.5	0.018	36.0	36.7	0.841
**Cormorbidity**						
**Charlson Comorbidity Index**	1 (0-2)	1 (0-2)	0.080	1 (0-2)	1 (1-2)	0.132
**COPD**	8.9	4.4	< 0.001	16.2	12.0	0.121
**Congestive heart failure**	7.3	11.2	< 0.001	7.3	9.1	0.393
**Cerebrovascular disease**	17.1	19.3	0.034	8.0	10.5	0.236
**Cancer**	14.6	11.0	< 0.001	22.2	23.3	0.736
**Principal diagnoses**			0.664			0.734
**Respiratory**	19.8	19.3		33.3	32.1	
**Non-respiratory**	80.2	80.7		66.7	67.9	
**Number of organ dysfunction**	1 (1-1)	1 (1-1)	0.779	1 (1-1)	1 (1-1)	0.284
**Hospital level**			0.085			0.905
**Medical center**	41.4	41.4		58.0	56.7	
**Regional hospital**	45.2	43.2		24.4	25.8	
**District hospital**	13.4	15.4		17.7	17.5	
**Types of mechanical ventilation**			< 0.001			0.001
**NIV-only**	2.3	4.3		32.5	42.5	
**NIV/INV**	9.3	9.7		1.1	3.3	
**INV-only**	88.3	86.0		66.4	54.2	
**Resource use**						
**Duration of MV**	3 (1-8)	3 (1-8)	0.831	1 (1-4)	2 (1-4)	0.954
**ICU LOS, days**	4 (2-9)	4 (2-9)	0.860	0	0	-
**Hospital LOS, days**	17 (8-30)	16 (8-29)	0.863	10 (5-19)	11 (6-19)	0.842
**Hospital mortality**	27.2	28.0	0.321	34.3	28.7	0.098

Values are expressed as median (interquartile range) or percentages.

COPD, chronic obstructive pulmonary disease; INV-only, use of invasive ventilation as the only ventilator treatment; LOS, length of stay; MV, mechanical ventilation; NIV-only, use of noninvasive ventilation as the only ventilator treatment; NIV/INV, use of both NIV and INV before or after each other.

Among ARF patients, women were 63% more likely than men to receive NIV-only after controlling for all potential confounders (Table 4). These differences changed little after using logistic GEE models, suggesting a low cluster effect. The results based on analyses of the propensity score matched pairs were similar (Table 4). The baseline characteristics of men and women were balanced after matching (Table 5). In subgroup analyses, the gender difference in the use of NIV-only was the highest for ARF patients with CHF, followed by those without COPD or CHF, but was insignificant for those with COPD (Table 4). The cluster effect on the use of NIV-only was unremarkable except in ARF patients without COPD or CHF because the gender difference became insignificant after using a logistic GEE model.

**Table 4:** Female-to-male odds of receiving different types of mechanical ventilation (MV) among patients with acute respiratory failure (ARF)*^a ^*

Types of MV	Conventional method^*b*^	Propensity score method^*c*^			
	**All ARF patients** **(M/F = 4177/2444)**	**ARF patients (M/F = 2390/2390)**	**ARF patients with COPD (M/F = 126/126)**	**ARF patients with CHF (M/F = 217/217)**	**Other ARF patients^*d *^(M/F = 2037/2037)**
	**aOR (95% CI)**	**aOR (95% CI)**	**aOR (95% CI)**	**aOR (95% CI)**	**aOR (95% CI)**

**Without GEE modeling^*a*^**					
**NIV-only**	1.63 (1.30-2.05)	1.50 (1.20-1.88)	1.08 (0.56-2.09)	2.76 (1.47-5.20)	1.43 (1.10-1.85)
**NIV/INV**	1.10 (0.91-1.32)	1.05 (0.86-1.28)	0.97 (0.50-1.86)	1.06 (0.58-1.92)	1.07 (0.85-1.35)
**INV-only**	0.76 (0.66-0.89)	0.80 (0.68-0.93)	0.97 (0.58-1.62)	0.55 (0.35-0.86)	0.81 (0.68-0.97)
**With GEE modeling^*a*^**					
**NIV-only**	1.61 (1.32-1.96)	1.58 (1.15-2.15)	1.07 (0.55-2.11)	2.76 (1.38-5.53)	1.43 (0.97-2.11)
**NIV/INV**	1.11 (0.96-1.29)	1.07 (0.85-1.35)	1.01 (0.48-2.14)	1.08 (0.57-2.03)	1.09 (0.82-1.45)
**INV-only**	0.75 (0.64-0.88)	0.77 (0.61-0.95)	0.95 (0.54-1.66)	0.55 (0.33-0.93)	0.80 (0.60-1.06)

*^a ^*Multivariable logistic regression models with and without considering the cluster effect of hospitals using GEE models.

*^b ^*Conventional multivariable logistic regression adjusting for age, surgical and selected comorbid conditions, Charlson Comorbidity index, hospital levels, principal diagnoses, number of acute organ dysfunction, and intensive care status.

*^c ^*Men and women were matched one-to-one by the propensity score and then analyzed in the logistic regression models.

*^d ^*Other ARF patients included those without underlying COPD or CHF.

aOR, adjusted odds ratio; CHF, congestive heart failure; CI, confidence interval; COPD, chronic obstructive pulmonary disease; GEE, generalized estimating equations; INV, invasive ventilation; INV-only, use of INV as the only ventilator treatment; NIV/INV, use of both NIV and INV before or after each other; NIV, noninvasive ventilation; NIV-only, use of NIV as the only ventilator treatment.

**Table 5:** Balances between men and women after one-to-one matching by the propensity score*

	ARF patients		ARF patients with COPD		ARF patients with CHF		Other ARF patients	
	**Women** **(*n *= 2390)**	**Men** **(*n *= 2390)**	**Women** **(*n *= 126)**	**Men** **(*n *= 126)**	**Women** **(*n *= 217)**	**Men** **(*n *= 217)**	**Women** **(*n *= 2037)**	**Men** **(*n *= 2037)**

**Age, years**	70 (55-80)	71 (56-79)	78 (72-85)	77 (70-83)	76 (70-82)	77 (68-82)	69 (53-79)	69 (53-78)
**Surgical condition**	52.9	53.6	22.2	21.4	28.6	33.2	57.2	57.8
**Comorbidity**								
**Charlson Comorbid Index**	1 (0-2)	1 (0-2)	2 (1-2)	1 (1-2)	2 (1-3)	2 (1-3)	1 (0-2)	1 (0-2)
**COPD**	5.4	5.2	-	-	11.5	12.9	-	-
**Congestive heart failure**	10.5	10.0	19.0	20.6	-	-	-	-
**Cerebrovascular disease**	18.2	18.9	13.5	11.9	7.8	5.5	19.6	21.1
**Cancer**	12.9	12.9	4.0	4.8	4.1	5.1	14.5	14.5
**Principal diagnoses**								
**Respiratory**	20.9	20.3	65.1	65.9	32.3	30.9	17.4	18.0
**Neurological**	1.7	1.6	8.7	10.3	0	0	1.8	1.9
**Cardiovascular**	24.7	24.5	12.7	10.3	48.8	49.3	22.4	22.4
**Digestive**	8.5	8.6	4.8	5.6	4.1	3.2	9.1	9.3
**Genitourinary**	2.8	2.5	4.0	3.2	2.3	3.2	2.8	2.2
**Metabolic/endocrine**	1.9	1.7	0	0	0	0	2.2.	1.7
**Injury/poisoning**	15.2	15.6	4.0	4.0	2.3	3.2	17.4	17.4
**Others**	24.4	25.3	8.7	10.3	10.1	10.1	26.8	27.0
**No. of organ dysfunction**	1(1-1)	1(1-1)	1(1-1)	1(1-1)	1(1-1)	1(1-1)	1(0-1)	1(0-1)
**Intensive care unit admission**	88.7	88.7	74.6	73.8	90.8	89.4	89.4	89.7
**Hospital level**								
**Medical center**	43.4	43.5	32.5	29.4	31.3	35.5	45.4	45.4
**Regional hospital**	41.2	41.1	42.9	47.6	47.0	47.0	40.4	40.9
**District hospital**	15.4	15.4	24.6	23.0	21.7	17.5	14.3	13.7

* Categorical variables are tested by the McNemar's test; continuous variables by the Wilcoxon Signed-rank test. All *P *values > 0.05.

Values are expressed as median (interquartile range) or percentages.

ARF, acute respiratory failure; CHF, congestive heart failure; COPD, chronic obstructive pulmonary disease; Other ARF, diseases other than COPD or CHF.

## Discussion

In this study, we found that women were more likely than men to receive NIV-only for ARF, especially those with underlying CHF or diseases other than COPD. We also confirmed the reports from western countries that women were less likely than men to receive intensive care and MV [1-3]. Despite these differences, hospital mortality in patients requiring MV or intensive care was similar between men and women.

The finding that women were more likely to receive NIV-only for ARF is consistent with prior reports [1-3]. Studies in Austria, Canada, Brazil, and the USA have shown that women are less likely than men to receive invasive treatments [1-3]. These findings suggest that ethnic, cultural, and geographical factors do not seem to contribute to the gender difference in the delivery of NIV for ARF. However, the social context of gender differences may influence the decision for initiating life-supporting treatment [1,2], which could possibly lead to a differential use of NIV. And there may be a mix of biological and clinical explanations for it. For example, a higher use of NIV in women may reflect their increased perception of breathlessness [16,17] and a lack of strict criteria for NIV use in clinical practices [9]. Therefore, women may receive NIV earlier, for longer, and perhaps more frequently because they may report more symptoms of dyspnea even under similar respiratory conditions [16,17]. Finally, the results of this study might also imply that women could be more likely to succeed in NIV treatment than men because the NIV-only group was mostly made of patients with NIV success (plus do-not-resuscitate patients who failed NIV without being intubated) and the NIV/INV group was mostly those with NIV failure (plus those who received a post-extubation NIV). Nevertheless, because gender is not shown to be associated with NIV failure [28], it might be more likely that a higher rate of NIV use in women simply reflects a gender bias in decision-making on NIV use instead of a higher rate of NIV success.

There are other reasons that might also explain some of the observed gender difference in NIV. First, misclassification between INV-only and NIV/INV was likely. In unselected patients with ARF receiving NIV, the reported intubation rates after a failed trial range from 10% to 60%; among them, 60% to 100% occur within 24 hours [8-10,13,29-33]. Therefore, the utilization rate of NIV in this study is likely to be underestimated. Because of the limitation in the Taiwan's NHI reimbursement policy (see methods section), the NHIRD tended to capture claims of patients receiving longer-duration NIV and misclassify those using INV-only and NIV/INV (i.e., to overestimate INV-only and underestimate NIV/INV). Since duration of NIV did not differ significantly between men and women receiving NIV/INV, misclassification would be similar for both genders and hence less likely to cause the observed difference. Besides, this bias cannot explain the difference observed in the provision of NIV-only. Second, some potential confounders could not be controlled in this study. For example, differential sex distributions have been reported in many aspects including the severity of acute illnesses [1], the occurrence of post-extubation stridor [34], the preference of advance directives (such as do-not-resuscitate or do-not-intubate orders) [35] and the population prevalence and incidence of some acute illnesses [36,37] or chronic comorbidities [38,39]; all of which have been shown to influence the outcome of, and hence the decision-making for, an NIV trial [28,39]. For this reason, we did a sub-analysis in propensity-score-matched pairs with underlying CHF and found that women were still more than twice as likely as men to receive NIV-only. And finally, a hospital cluster effect may also explain some of the observed difference in subgroups such as ARF patients with diseases other than COPD or CHF.

The reasons that the gender difference in the provision of NIV was not found in patients with COPD are unclear. As COPD is the most well-established disease benefited from NIV [4,5], the gender difference may be less likely to occur. However, because women are at risk of under-diagnosis, and hence being under-coded, of COPD [40], the results could be either over- or under-estimated. For example, failure to code for COPD would hinder the adjustment for women and thereby bias the observed association between women and NIV away from the null. On the other hand, failure to diagnose COPD could theoretically result in under-utilization of NIV in women. This may also explain the null finding in ARF patients with COPD.

Our study provides the first nationwide population-based data on the utilization rate of NIV (including NIV-only and NIV/INV) both outside and inside the ICU in Taiwan. The utilization rate was slightly lower than that in developed countries (15.4% vs. 16% to 24%, respectively) [12-14,29,41]. The reported rates in developed countries are also likely to be under-estimated by including only ICU patients [12-14,30,41]; and the under-estimation may become more significant over time due to increased successful use of NIV outside the ICU [14,42,43]. For example, in a regional survey of 82 acute care hospitals in the USA, 20% of ARF patients received NIV, of which 45% of the treatment was initiated outside the ICU [42]. In this study, 27.7% of patients receiving NIV were cared for only outside the ICU.

The effect of gender on the outcome of patients requiring intensive care or MV remains debatable [1,2,31,44-46]. Although a higher risk of death in women requiring intensive care or MV has been found in some studies [2,44,45], it is not confirmed by others [1,31,46]. Our study did not find the gender effect on the outcomes of these patients despite the observed difference in the process of care. This finding is consistent with a prior report [1] showing that a more invasive therapeutic approach in men does not translate to a better outcome.

The finding of a hospital cluster effect on the gender difference in NIV use may provide policy implications for best practice provision across hospitals. The cluster effect implies that clinicians are not using standard criteria to start NIV based on evidence from guidelines [5,12]. Non-adherence to guidelines is also present in situations eligible for NIV, leading to its underuse [12,47]. For example, one retrospective study showed that nearly two thirds of ICU patients with exacerbation of COPD or CHF did not receive an NIV trial despite meeting eligibility criteria [47]. However, because the indication for NIV could not be determined in this study, we do not know whether the use of NIV for ARF is appropriate to the guidelines.

Several other limitations deserve comments. First, the administrative databases are subject to possible under-coding and over-coding errors. The definitions of diagnoses relied solely on diagnostic codes, but the accuracy of which could not be verified. Second, information on primary causes of ARF, indications/mode/settings and effectiveness of NIV use, and types of ventilators was not available. Third, hospital mortality might be under-estimated for the inability to verify through linkage to death certificate. And finally, since only five diagnostic codes were available, some related diagnoses could have been missed. But these biases are toward the null. Nevertheless, our study is strengthened by the large number of patients retrieved from a nationwide population-based dataset, which can provide an unbiased selection and enhance its generalizability.

## Conclusions

This study suggests that gender difference not only existed in the provision of intensive care and MV, but also in the use of NIV in Taiwan. The subgroup analyses indicate that the gender differences in NIV use were heterogeneous and related to the underlying diseases. Further research is needed to explore why gender differences exist, especially whether and how gender-biased decision-making affects the use of NIV.

## Key messages

• Studies in western countries have shown that gender differences exist in the process of care for critically ill patients.

• Our findings confirm the reports from western countries that women were less likely than men to receive intensive care and mechanical ventilation.

• Moreover, we found that women were more likely than men to receive NIV-only for acute respiratory failure, especially those with underlying heart failure.

• The finding of a higher NIV use in women may reflect a higher perception for dyspnea and their lower tendency to receive invasive treatments than men as well as a lack of strict criteria for NIV use in clinical practices.

• Further research is needed to understand why the gender difference occurs, especially whether and how gender-biased decision-making affects the use of NIV.

## Abbreviations

aOR: adjusted odds ratio; ARF: acute respiratory failure; CHF: congestive heart failure; CI: confidence interval; COPD: chronic obstructive pulmonary disease; GEE: generalized estimating equations; *ICD*-9-CM: *International Classification of Diseases*: Ninth Revision: Clinical Modification; INV: invasive ventilation; IQR: interquartile range; LHID: Longitudinal Health Insurance Database; MV: mechanical ventilation; NHIRD: National Health Insurance Research Database; NIV: noninvasive ventilation.

## Competing interests

The authors declare that they have no competing interests.

## Authors' contributions

HNS designed the study, obtained funding, performed data mining and processing, did statistical analyses, drafted the initial manuscript, and revised important content. CLL contributed to the study design, data mining and processing, analyses and interpretation of results, and revision for important content. HHY participated in the interpretation of results and revision for important content. All authors read and approved the final manuscript.
